# The Application of Induced Pluripotent Stem Cells Against Liver Diseases: An Update and a Review

**DOI:** 10.3389/fmed.2021.644594

**Published:** 2021-07-01

**Authors:** Lei Zhang, Ke Pu, Xiaojun Liu, Sarah Da Won Bae, Romario Nguyen, Suyang Bai, Yi Li, Liang Qiao

**Affiliations:** ^1^The First Clinical Medical College, Lanzhou University, Lanzhou, China; ^2^Department of General Surgery, The First Hospital of Lanzhou University, Lanzhou, China; ^3^Key Laboratory of Biological Therapy and Regenerative Medicine Transformation Gansu Province, Lanzhou, China; ^4^Department of Gastroenterology, The First Hospital of Lanzhou University, Lanzhou, China; ^5^Key Laboratory for Gastrointestinal Diseases of Gansu Province, Lanzhou University, Lanzhou, China; ^6^Department of Medical Oncology, The First Hospital of Lanzhou University, Lanzhou, China; ^7^Storr Liver Centre, Westmead Institute for Medical Research, University of Sydney at Westmead Clinical School, Westmead, NSW, Australia

**Keywords:** HLCs, primary human hepatocytes, liver disease, clinical application, IPS cell

## Abstract

Liver diseases are a major health concern globally, and are associated with poor survival and prognosis of patients. This creates the need for patients to accept the main alternative treatment of liver transplantation to prevent progression to end-stage liver disease. Investigation of the molecular mechanisms underpinning complex liver diseases and their pathology is an emerging goal of stem cell scope. Human induced pluripotent stem cells (hiPSCs) derived from somatic cells are a promising alternative approach to the treatment of liver disease, and a prospective model for studying complex liver diseases. Here, we review hiPSC technology of cell reprogramming and differentiation, and discuss the potential application of hiPSC-derived liver cells, such as hepatocytes and cholangiocytes, in refractory liver-disease modeling and treatment, and drug screening and toxicity testing. We also consider hiPSC safety in clinical applications, based on genomic and epigenetic alterations, tumorigenicity, and immunogenicity.

## Introduction

Liver disease causes ~2 million deaths annually worldwide. Cirrhosis-related complications account for 50% of deaths, and viral hepatitis and hepatocellular carcinoma (HCC) together account for the other 50% of deaths annually worldwide. Cirrhosis is currently the 11th most common cause of death globally, and liver cancer is the 16th leading cause of death. Cirrhosis and HCC together account for 3.5% of all deaths worldwide (1). Currently, liver transplantation is the most effective treatment option for patients with end-stage liver disease (ESLD). However, <10% of global liver transplantation needs are met (2). The shortage of donor organs and transplant costs are the major limiting factors (3). In addition, the recipient's immune system may reject the transplanted organ (4). Therefore, the development of alternative therapeutic strategies for patients with chronic liver disease is of utmost importance.

Human induced pluripotent stem cells (hiPSCs) are pluripotent stem cells that are induced and reprogrammed from adult cells into undifferentiated cells by exposure to specific mediators. They have the capacity for self-renewal and differentiation into a variety of somatic cells, such as embryonic stem cells (5). HiPSC-derived hepatocyte-like cells (hiPSC-HLCs) exhibit morphological and phenotypic characteristics of primary human hepatocytes (PHHs) (6). hiPSC-HLCs should be incorporated into future studies of liver diseases to solve the problems of organ shortage and prevent recipient immunorejection of PHHs. hiPSC-HLCs can be considered as an ideal source of hepatocytes. In addition, these cells may enable cell-based therapy, exploration of liver disease models, *in vitro* models for pharmacology and toxicology studies (7) and ESLD treatment.

In this review article, we aimed to provide a perspective on the current organization and provision of transplant services based on specific challenges and environmental settings. We review how these specific requirements are addressed by the existing cell culture systems for the generation of hiPSC-derived hepatocytes, describe their applications for modeling hepatic disorders, and discuss future directions for the use of hiPSCs in the study and treatment of liver diseases.

## Recent Developments in the iPSC Reprogramming Methodology

Protocols for somatic cells transformed into induced pluripotent stem cells are widely used for iPSC reprogramming method. The key to the success of these protocols is the ability to efficiently induce pluripotent cells to adopt a definitive endoderm fate. Earlier studies have shown that mouse fibroblasts can be reprogrammed to develop embryonic stem cell (ESC)-like features, and grow in the presence of four factors: Oct3/4, Sox2, c-Myc, and Klf4 (8). These induced cells were named iPSCs. These insights opened the door to further refinements in the cell differentiation methodology and enabled the derivation of visceral, endodermal-derived tissues. The same protocol was used to successfully generate iPSCs from adult human skin cells (9). In addition, human somatic cells were transformed into PSCs by inducing the expression of a new set of four factors: Oct4, Sox2, Nanog, and Lin28 (10). Many optimization steps have been devised to improve the efficiency of reprogramming factors, such as the use of integration-free methods (8, 11). Episomal vectors, Sendai viruses, and synthetic mRNAs are among the most commonly used methods for generating hiPSCs (12–14) without modifying the host genome, which could interfere with disease modeling or experimental outcomes.

## Current Approaches for Generation of hiPSC-HLCs and Cholangiocyte-Like Cells

**Figure 1:**
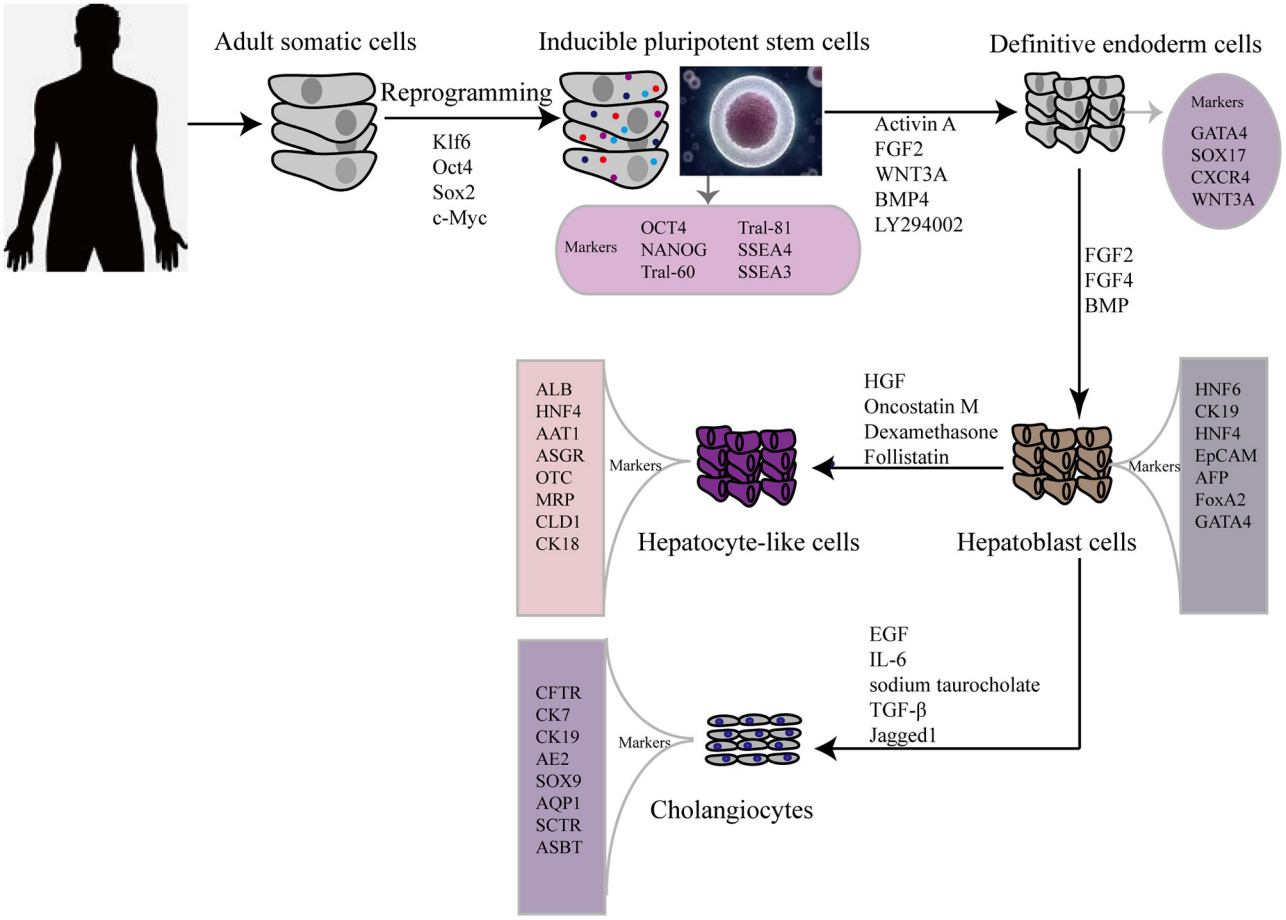
Flow diagram showing typical protocol for iPSCs reprogramming and hepatic differentiation of human into hepatocyte-like cells *in vitro*. OCT 4, octamer binding transcription factor 3/4; SSEA, stage-specific embryonic antigen; TRA, Tumor resistance antigen 1-60; SOX2 sex determining region Y box 2; KLF4 (Kruppel-like factor 4); GATA4, GATA binding protein 4; CXCR4, C-X-C chemokine receptor type 4; FGF, fibroblast growth factor; HGF, hepatocyte growth factor; BMP, bone morphogenetic protein; HNF6, Hepatocyte nuclear factor 6; CK, cytokeratin; EpCAM, Epithelial cell adhesion molecule; AFP, alpha-fetoprotein; AAT1, α1-anti-tripsin; ALB, albumin; ASGR, asialoglycoprotein receptor; MRP, multidrug resistance protein; CLD, claudin; EGF, epidermal growth factor; IL-6, interleukin 6; TGF-β, transforming growth factor beta; CK7, cytokeratin 7; CK19, cytokeratin 19; AE2, chloride/bicarbonate anion exchanger 2; ASBT, apical sodium-dependent bile acid transporter; CFTR, cystic fibrosis transmembrane conductance regulator; AQP1, aquaporin-1; SOX9, SRY-box 9; SCTR, secretin receptor.

In 2009, hiPSCs were induced to differentiate into hepatic cells for the first time, via a timed administration of various growth factors. The expression of hepatocyte markers and liver-related functions in hiPSC-HLCs was monitored and compared with those in differentiated human ESCs and PHHs (15). This revealed that hepatic cells could be generated from iPSCs but the process took more than 20 d. To overcome this, the differentiation step was revised to a more efficient three-step protocol, allowing rapid generation of HLCs from hiPSCs (Figure 1) (16). Hepatic progenitor-like cells derived from hiPSCs possess the potential for bipotent differentiation into HLCs and cholangiocyte-like cells (17).

For clinical applications, differentiated cells should be assessed by comparing them with primary liver-derived cells, to verify their morphology and expression of liver-specific proteins, such as alpha-fetoprotein (AFP) and albumin. However, neither ESCs nor iPSCs can differentiate into fully mature hepatocytes *in vitro*. As a consequence of an emerging interest in using iPSCs in regenerative medicine to treat liver diseases, many researchers are focused on developing the most efficient and reproducible approaches for the derivation of high-quality hiPSC-HLCs. Currently, the differentiation strategies for obtaining hiPSC-HLCs generally require a stepwise induction of definitive endoderm, hepatocyte specification, and hepatoblast expansion into mature HLCs. Activin A, Wnt3a (18)and fibroblast growth factor 2 (FGF) signaling (19) play important roles in hiPSC differentiation toward hepatic endoderm, whereas hepatocyte growth factor (HGF) promotes the growth of hepatoblast cells (20, 21). Another factor, the interleukin (IL) 6 family cytokine oncostatin M (OSM), combined with the glucocorticoid dexamethasone (DEX), accelerates the maturation of hepatocytes. Meanwhile, many protocols for iPSC differentiation to hepatocytes have been reported, which involve the use of various growth factors and cytokines, plating techniques, and transduction of key liver-specific transcription factors (22).

Other studies have reported strategies for the generation of cholangiocyte-like cells from hiPSCs. The first differentiation of cholangiocyte-like cells from hiPSC hepatoblasts was induced in the presence of growth hormone, epidermal growth factor (EGF), IL-6, and sodium taurocholate (23). Subsequently, efficient differentiation of cholangiocytes from iPSCs was improved by 3D co-culture of hepatoblasts and OP9 stromal cells in the presence of HGF, EGF, and TGF-β (24), and by stimulating cholangiocyte progenitor specification using FGF10, activin A, and retinoic acid (25). Yet another stepwise cholangiocyte differentiation approach involves a definitive endoderm–hepatic specification–hepatic progenitor–cholangiocyte procedure, with Jagged1 and TGF-β supplementation being key for the promotion of iPSC-cholangiocyte formation (26). These cells were subsequently characterized *in vitro* and *in vivo*. Induced-cholangiocytes show mature markers, such as SOX9, CK7, CK19, CFTR, AE2, ASBT, AQP1, and SCTR and they are negative for the hepatocyte marker HNF4a.

## Features and Functions oF hiPSC-HLCs

**Figure 2:**
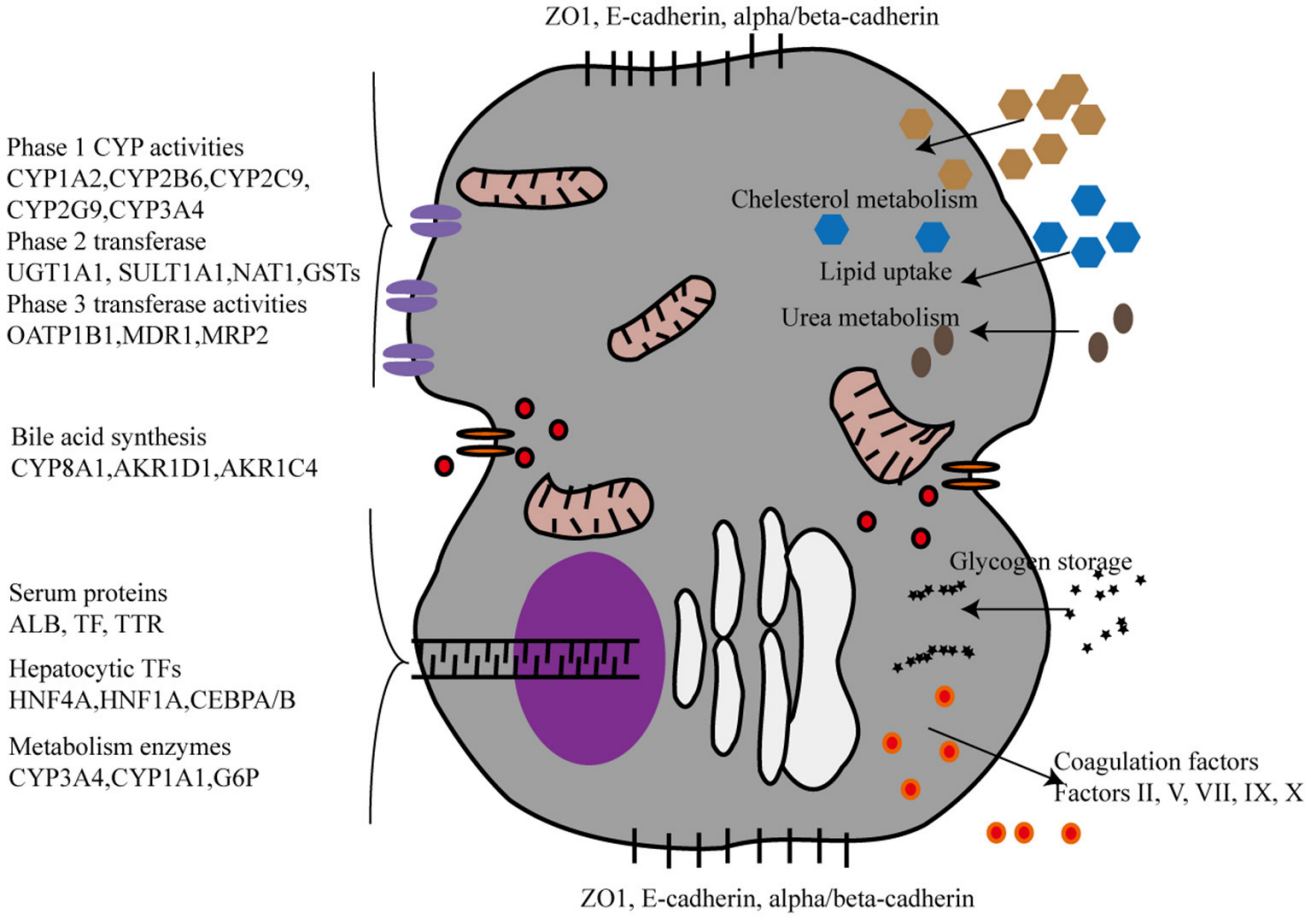
The phenotypic characterization of Hepatocyte-like cells derived from iPSCs. CYP, CYPs cytochromes P450; UGT, UDP glucuronosyltransferase. SULT1A1, Sulfotransferase Family 1A Member 1; NAT1, N-Acetyltransferase 1; GSTs, Glutathione S-Transferase; OATP, organic anion-transporting polypeptide; MDR, Multiple drug resistance; MRP2:multidrug resistance protein 2; AKR1D1, aldo-keto reductase family 1 member D1, AKR1C4, aldo-keto reductase family 1 member C4, TTR:Transthyretin, TP, Total protein, ALB, Albumin, HNF, Hepatocyte nuclear factor, CEBPA/B, CCAAT Enhancer Binding Protein Alpha/Beta, G6P, Glucose-6-Phosphatase.

The liver performs a wide range of fundamental functions, including metabolic, nutrient storage, and detoxification functions. Hence, each protocol for an efficient maturation of iPSCs into HLCs needs to incorporate a thorough and critical evaluation of the hepato-specific transcriptome and enzymatic activities of the obtained cells. Drug metabolizing capacity, urea cycle activity, or bile acid and lipoprotein synthesis and excretion need to be evaluated to ascertain HLC maturation (Figure 2). By contrast, most of the current reports on HLC generation show that HLCs express genes at levels that are characteristic for the fetal liver and display a fetal-like phenotype. According to one study (26), HLC mitochondria display specific morphological changes, such as elongation, swollen cristae, dense matrix, and cytoplasmic migration, with an increased expression of mitochondrial DNA transcription and replication-related genes, and increased oxygen consumption. Following differentiation, HLCs express liver-specific proteins, including albumin and hepatocyte nuclear factor 4 alpha, and show intrinsic hepatocyte functions, including CYP450 activity. However, HLCs also express high levels of AFP, suggesting a persistent immature phenotype or inability to turn off early-stage genes. Furthermore, albumin production, urea production, CYP450 activity, and mitochondrial function of HLCs are significantly lower than those of primary human hepatocytes. Functional indicators, urea synthesis, glycogen synthesis, lipid storage, indocyanine green intake, and low-density lipoprotein intake, are also used to evaluate the function of HLCs (27–31) (Table 1). Liver function after HLC transplantation in disease-specific models in mouse is assessed using glycogen content synthesis, low-density lipoprotein, and indocyanine green intake (32).

**Table 1:** Functional features of hepatocyte-like cells (HLCs) derived from human induced pluripotent stem cells.

**Function features**	**hiPSC-HLCs**	**PHHs**
Phase I CYP activities		
CYP1A2 (28, 31)	–	+
CYP2B6 (28, 31)	–	+
CYP3A4 (31)	–	+
CYP3A7 (31)	+	–
Phase II transferase (27)	+	+
UGT1A1	+	+
SULT1A1	+	+
NAT1	+	+
GSTs	+	+
Phase III transferase (27)	+	+
OATP1B1	+	+
MDR1	+	+
MRP2	+	+
Hepatocytic TFs	+	+
HNF4A (29, 30)	+	+
AFP (27, 29)	+	–
Bile acid synthesis	+	+
Albumin synthesis (29, 30)	+	+
Glycogen storage (30)	+	+
LDL uptake (29, 30)	+	+
Urea metabolism (30)	+	+
Cholesterol metabolism (29)	+	+

Hepatocyte isolation is not easy to perform, and requires expertise and access to primary human liver tissue. An alternative strategy for the acquisition of hepatocytes may grant the researchers access to important control material, thanks to commercially available cryopreserved hepatocytes, or as an alternative to using human liver tissue. Recently, an important and detailed report describing the expression of more than 60 hepatic, pluripotency, and developmental genes has been published to map their changes during liver cell isolation and stem cell maturation into HLC (33). In this report, the specific traits of iPSC-HLC were compared with those of adult liver and fetal liver cells, proving that several hepatic enzymes, whose expression is limited during the fetal period and absent at the postnatal stage, are highly expressed in iPSC-HLCs. In the study, *CYP3A4* and *CYP3A7* genes were used for a convenient and quick evaluation of hepatic maturation.

## hiPSC-HLCs for the Treatment of ESLD

At present, some promising stem cell-based transportation treatments for ESLD have been reported. Several studies have demonstrated the restoration of liver function in hematopoietic stem cells (HSCs), mesenchymal stem cells (MSCs), and endothelial progenitor cells (EPCs) in acute liver failure (34–37). Although alleviation of liver dysfunction was observed in patients who have received these treatments in clinical trials, the safety and short-term efficacy of stem cell-based transplantation were not explored in detail and should be further evaluated in large-scale prospective cohort studies (38). In fact, stem cell transplantation as an alternative treatment for ESLD cannot be used for remodeling injured hepatic structure. In theory, iPSC-HLCs can be regarded as the optimal cell replacement treatment for acute liver failure (ALF) and ESLD. Meanwhile, HLCs derived from patient-specific iPSCs are an unlimited source of hepatocytes to treat liver failure.

To date, the application of iPSCs in ALF and liver failure has been tested in animal models. Indeed, iPSC-HLC transplantation improves the general condition in a mouse model of CCl_4_-induced liver injury (39). Further, the survival rate of immunodeficient mice with ALF significantly increased and liver fibrosis levels in mice with chronic liver injury significantly decreased after hiPSC-HLC transplantation (40). According to another study, hiPSC-HLCs rescue drug-induced ALF in rodents, and hiPSC-HLCs have functional and proliferative potential for liver regeneration after transplantation in an ALF model (41, 42). These studies indicated the potential of these cells in regenerative medicine for future clinical applications. Of note, the aforementioned studies only focused on rodent models rather than primates. To date, no studies have illustrated the application of iPSC-HLCs in human liver failure.

The first transplantation of hiPSCs in regenerative medicine took place in 2014, and was performed to treat a 77-year-old female patient with polypoidal choroidal vasculopathy in both eyes. Autologous iPSCs were generated from the patient's skin fibroblasts, differentiated into retinal pigment epithelium, and then transplanted into the eye. Immune response to autologous transplantation was observed 18 months postoperatively, and the results indicated no complications. The retinal pigment epithelial sheet that had been transplanted survived well, and the corrected visual acuity of the treated eye did not improve or worsen.

Unfortunately, not all cases are as successful as the autologous transplant discussed above. Another clinical trial involving three patients with age-related macular degeneration involved intravitreal administration of stem cells derived from autologous adipose tissue. Post-treatment observation revealed vision loss associated with ocular hypertension, hemorrhagic retinopathy, vitreous hemorrhage, combined traction and rhegmatogenous retinal detachment, or lens dislocation (43).

The above trials demonstrate the need for further investigation of stem cell therapy and, more specifically, the use of hiPSCs in regenerative medicine to treat ESLD.

## hiPSC-HLC Modeling of Liver Disease Development

### Infectious Liver Diseases

Hepatitis B virus (HBV) and hepatitis C virus (HCV) are the most prevalent agents of infectious liver diseases. Approximately 520 million people suffer from infections caused by one of these viruses worldwide, including 170 million people with HBV and 350 million people with HCV infections (44). The infected population will develop virus-related cirrhosis and liver cancer (45). At present, the interaction between these viruses and host hepatocytes remains unclear because of the absence of viral culture models to reflect the infection process and reproduce the molecular events within an infected host cell.

PHHs are the gold standard for studying the physiopathology of liver infections. However, low cell viability and yield caused by a rapid loss of hepatic phenotype upon isolation from the liver microenvironment are the main limitations of their application. As an alternative to overcome these limitations, iPSC-HLCs could be used to study the viral infection process and virus–host interactions, as well as the viral life cycle, to ultimately identify efficacious drugs for infectious liver diseases.

In the past, iPSC-HLCs have been successfully used as an *in vitro* system for modeling hepatitis virus infections and virus–host interactions. When HCV entry and genomic replication is stimulated, iPSC-HLCs more commonly express HCV receptors and show increased susceptibility to HCV infection than PHHs (46). Further, HCV entry inhibitor (CD81 antibody) and HCV genomic replication inhibitor (interferon) attenuate HCV pseudovirus entry and HCV sub-genomic replication, respectively, in iPSC-HLCs. It has been reported that hiPSC-HLCs support the entire life cycle of HCV, including the inflammatory response to infection and host genetics impacting viral pathogenesis (31). Furthermore, iPSC-HLCs that show an appropriate antiviral response produce interferon (47, 48) and survive *in vitro* for up to 1 week after inoculation with HCV (47), comprising a superior model for observing hepatocyte function during a relatively long-term infection. Detailed molecular mechanisms allowing viral infection can also be investigated using this model (49).

The above observations suggest that iPSC-HLCs can act as a promising cell model for analyzing hepatocyte responses to viral infection, as well as an ideal platform for drug target discovery for HCV therapy. However, some limitations should be considered. For example, some studies have reported lower virus titers in culture supernatants of HBV-infected iPSC-HLCs than those in PHHs (50), and suggested lack of functional maturation of iPSC-HLCs obtained using various differentiation protocols. Finally, it is necessary to increase the diversity of hiPSC lines used in similar such analyses to assess the impact of the host genetic background on the cellular response and efficiency of infection.

### Inherited Metabolic Disorders of the Liver (IMDs)

The liver is vital for metabolic homeostasis. Approximately 70% of patients with IMDs are affected by liver tissue damage. α1-Antitrypsin deficiency (AATD) and familial hypercholesterolemia (FH) are common IMDs that have been extensively studied. AATD results from a single base-pair mutation (leading to Glu342Lys substitution in the protein product), known as the Z mutation, in the *SERPINA1* gene. The substitution causes the protein to misfold and be retained in the endoplasmic reticulum (ER), with the formed protein polymers inducing hepatocyte death (51). By contrast, FH is an autosomal dominant hypercholesterolemia caused by mutations in a gene for the low-density lipoprotein receptor (LDLR) or LDLR-related genes. FH is characterized by elevated serum levels of low-density lipoprotein (LDL)-cholesterol (C), which lead to xanthoma formation and premature cardiovascular disease (52, 53).

The use of iPSC-HLCs as a novel model of the above monogenic diseases and iPSC-HLC application for a gene correction therapy for the generation of disease-free autologous cells are attracting increasing attention. For instance, accumulation of A1AT variant polymers in the endoplasmic reticulum of iPSC-HLCs was observed when iPSCs from AATD patients were differentiated into HLCs (54). Further, biochemical features and morphological manifestations of iPSC-HLC models generated from cells from AATD patients with and without severe liver disease (SLD) were explored (55). The analysis of individual disease phenotypes of AATD patients revealed rapid degradation of misfolded α1-antitrypsin Z (ATZ) and no globular inclusions in cells from patients in which the liver disease had been ameliorated (55). Similar, the main pathological characteristic of FH was recapitulated by inducing iPSCs obtained from patients with FH to form HLCs (54). In addition, analysis of iPSC-HLCs obtained from an FH patient with a mutation in the *LDLR* gene demonstrated that FH-derived iPSC-HLCs are unable to take up LDL-C, and secrete more apolipoprotein B-100 than the controls (56). Furthermore, these HLCs do not respond to statin treatment (56). Together, these observations demonstrate that IMD-hiPSCs effectively model the pathological features of IMD.

Animal models have been used to assess the efficacy of iPSC-HLC therapy in IMDs (57). The first reported targeted gene correction of AATD in iPSCs was achieved by bi-allelic correction of mutated loci by using zinc finger nucleases (ZFNs) and PB technology to correct the *A1AT* gene in hiPSCs (58). Genetic correction of iPSC-HLCs restored the normal structure and function of the A1AT protein *in vitro* and *in vivo*. Gene correction for AATD has been also successfully attempted using the TALEN approach, which is more efficient than ZEN (59). In addition, the use of CRISPR/Cas9 gene editing technology to correct point mutations in specific genes has been reported (60) and experimental data have highlighted the advantages of using the CRISPR/Cas9 system for allele-specific genome targeting and for gene disruption mediated by non-homologous end joining. Indeed, CRISPR/Cas9 genome editing was used to permanently correct a 3-bp homozygous deletion in *LDLR* exon 4 in patient-derived homozygous FH (HoFH)-iPSCs (61). This genetic correction restored LDLR-mediated endocytosis in FH-HLCs and is a proof-of-principle that CRISPR-mediated genetic modification can be successfully used to normalize HoFH cholesterol metabolism deficiency at the cellular level. The above findings clearly demonstrate that genome-editing technology can be used to achieve functional correction of patient-derived iPSC-HLCs.

### Non-alcoholic Fatty Liver Disease (NAFLD)

NAFLD is becoming a serious clinical concern because of its severe morbidity and potential progression to ESLD, such as liver cirrhosis and HCC (62). The current global prevalence of NAFLD is estimated to be 25.24% (63) and NAFLD is the second most common cause of liver transplantation (64). The disease develops when chronic hepatic lipid accumulation stimulates an overload of metabolic alterations, including mitochondrial dysfunction, endoplasmic reticulum stress, and hepatic insulin resistance, and induces an inflammatory response (65–68). It is not possible to predict the progression of NAFLD because of adverse events and sampling variability of the currently used invasive diagnostic methods (liver biopsy) (69).

Several studies have reported cell models of NAFLD that use HCC cell lines or immortalized primary hepatocytes (70, 71) Differences in cell function and gene expression between the two have been demonstrated. However, the main limitation of these models is that cultivation of liver biopsy-derived primary hepatocytes for NAFLD modeling takes several days. Hence, using iPSC-HLCs to model NAFLD and non-alcoholic steatohepatitis (NASH) would facilitate research on molecular diagnosis and prognosis, disease progression, and drug development for NAFLD. hiPSC-HLCs have been used as an *in vitro* model to first demonstrate intracellular lipid accumulation in NAFLD (72). Major changes in the expression of metabolism-associated genes and upregulation of the lipid droplet-coating protein Perilipin2 (PLIN2) were detected in the model. Upregulation of the expression of numerous genes of the peroxisome proliferator-activated receptor (PPAR) pathway, constituting a regulatory hub for metabolic processes, was also detected. Taking previous studies into consideration, an iPSC-HLCs model with the *PNPLA3* genotype that is closely associated with hepatic steatosis in moderate (30–40% fatty changes) and severe (70% fatty changes) NAFLD patients was established and characterized (73, 74). Other studies, on the association between endoplasmic reticulum stress response and hepatocyte metabolism disorders in hiPSC-HLCs have been published (75). ER stress pathways play an important role in lipid metabolism, and ER stress enhances lipid accumulation ~5-fold in hiPSC-HLCs compared to their respective controls (71).

Taken together, the hiPSC-HLC model of NAFLD can be used to characterize some of the metabolic features of NAFLD. However, the pathological progression of lipotoxicity in NAFLD involves not only lipid accumulation but also a complex pathophysiological response, such as inflammation and the immune response. Future studies should highlight the applicability of hiPSC-HLCs as a discovery platform for the exploration of molecular events and aid in drug development for NAFLD.

### Liver Cirrhosis

Liver cirrhosis is characterized by the presence of diffuse, chronic necro-inflammatory, and fibrogenetic hepatocytes, ultimately leading to the development of features of chronic liver injury, such as structurally abnormal nodules, dense fibrotic septa, concomitant parenchymal exhaustion, and collapse of the liver tissue (76). Chronic liver injury remains one of the most common causes of death in the Western world (77). Currently, modeling liver cirrhosis *in vitro* to unveil the intricate cellular interactions underlying its pathological development is challenging. Traditional approaches involve treatment of cell culture models with toxic drugs or compounds that are often not specific to liver disease (78). Meanwhile, 3D models of primary hepatocyte co-culture with stellate cells are limited by the availability of tissue samples (79). 3D-Organoid cell culture models using iPSC-HLCs, stromal cells, and Kupffer cells (80, 81) can be an excellent approach to study the cellular interactions underlying the pathophysiology of liver cirrhosis. However, some chronic liver injuries, such as cardiac cirrhosis, are difficult to study in an *in vitro* setting and require major advances in 3D co-culture systems.

Previous studies have demonstrated enhanced liver regeneration in mouse model (42), reduced murine liver fibrosis (82) and stabilization of chronic liver disease (39) following iPSC-HLC administration, highlighting its potential as a therapeutic strategy for liver cirrhosis. Recently, the anti-fibrotic properties of iPSCs were demonstrated. Namely, iPSC-derived extracellular vesicles (EVs) were shown to regulate hepatic stellate cell activation and have anti-fibrotic effects (83). Consequently, the use of iPSC-EVs is regarded as a novel anti-fibrotic approach that may reduce or reverse liver fibrosis in patients with chronic liver disease. Furthermore, the therapeutic potential of iPSC-HLCs in liver fibrosis was explored by generation of iPSC-HLCs from mouse embryonic fibroblasts by using a reprogramming technology, and migrating iPSC-HLCs cluster to the intra-spleen and the liver (84). Transplantation of iPSC-HLCs significantly attenuated liver fibrosis induced by CCL4 (80); hence, iPSC-HLCs may be used as a novel therapeutic strategy for the treatment of liver fibrosis. However, while a cell-based therapy for cirrhosis can temporarily relieve cellular hepatocyte injury, it cannot eliminate collagen deposits or restore the original liver structure. Further detailed studies could bring about a novel method by which fibrogenic cells can be reprogrammed into hepatic parenchymal cells in the cirrhotic liver (85).

### Liver Cancer

HCC is the third leading cause of cancer-related deaths worldwide and the sixth most common malignancy (86, 87). Liver cancer research mainly focuses on the molecular pathways and treatments for HCC. Within the cancer stem cell (CSC) research, previous reports have explored the possibility of generating liver CSCs by the induction of reprogramming-related factors, such as Oct4 or Nanog (88, 89). However, the induction of HCC cells into liver CSCs using pluripotency-related transcription factors has not yet been widely studied. According to one report, reprogramming can be achieved in tumor cells by retroviral induction of reprograming-associated genes (76). Interestingly, the reprogrammed pluripotent cancer cells (iPCs) were very different from the original cancer cells in terms of colony shape and gene expression of tumor markers. Further, the induction of pluripotent liver cancer cells is correlated with the p53 status, suggesting that varying the gene expression level of p53 may affect the reprogramming process.

In one study, potential tumorigenicity of hiPSC-HLCs during differential induction from hiPSCs to HLC was observed after knockdown of p21 (90). The authors of that study also investigated whether hepatoma-like cells derived from hiPSCs of HCC patients can be transformed into normal hepatocyte cells upon treatment with acyclic retinoid and AKR1B10 inhibitor (tolestat). Combining acyclic retinoid (10 μM) with tolestat (10 μM) is considered to be an appropriate regimen for inducing differentiation of hepatoma-like cells into hepatocytes. The efficacy and toxicity of this combination therapy for individual patients with HCC will be evaluated in the near future.

Previously, iPSCs have been used to treat HCC. More specifically, the therapeutic effect of IPS cell-derived myeloid lineage cells (iPS-ML) and their ability to produce interferon (IFN) β in primary and metastatic liver cancer were reported in mice xenograft model of liver metastasis (91). Further, iPS-ML producing IFN-β injection hindered cancer progression and increased the survival rate in a mouse model.

In summary, at present, the applicability of iPSC technology for HCC treatment mainly focuses on cell reprogramming from HCC to CSCs, and the search for novel HCC treatments. The above studies provide valuable insights for studying and treating HCC.

### hiPSC-Cholangiocyte Modeling of Cholangiocyte Disease Development

Cystic fibrosis (CF) is a single-gene inherited disease characterized by mutations in the cystic fibrosis transmembrane conductance regulator gene (*CFTR*), affecting the function of chloride ion channels, with intrahepatic bile stasis. The iPSC-cholangiocytes are superior to other cells from the perspective of the exploration of these diseases and drug discovery. In 2015, two studies reported generation of cholangiocyte organoids from CF patient-iPSCs using their iPSC-cholangiocytes protocols (24, 92). The disease phenotype was modeled in these studies by exploring non-functional CFTR proteins, with subsequent chloride channel impairment and the inability of fluid secretion to form cysts. The effects of the drug VX809 on CF were also tested, demonstrating a functional rescue of impaired CFTR proteins. Both disease models illustrate valid applications of iPSCs, not only as a proof of pathophysiological interactions, but also for biliary-specific pharmacological screening.

Currently, no other types of cholangiopathy have been modeled using iPSC-cholangiocytes, modeling these disease-types rely on animal models of a deficiency or mutation of disease-specific genes. It may be easier to characterize disease pathophysiology and obtain detailed drug test information if cholangiocytes derived from patient-related iPSC have direct disease-specific genes information. The etiology of biliary atresia, primary biliary cholangitis, primary sclerosing cholangitis, and cholangiocarcinoma is still unknown because of the intricate interaction between the environment and the genes. Therefore, iPSC cholangiocytes derived from disease-specific cases can be studied to confirm the etiological hypotheses and identify potential therapeutic targets.

## Drug Discovery and Toxicity Testing

Another important application of iPSCs is assessing the therapeutic effect and toxicity of drugs, as has been reported in many studies (93, 94). The effect of drugs is influenced by the genetic background and other complex factors. It is easier to screen drugs using iPSCs than via pharmacological testing of animals. Consequently, organoids, as an innovative technique for drug screening and toxicity assessment, have been extensively researched. However, iPSC-derived organoids in 2D culture cannot be used for drug screening because iPSCs in such culture receive similar stimuli as those in monolayer and cannot be used to model the physiological microenvironment. By contrast, 3D culture models the *in vivo* microenvironment and approximates the physiological conditions. Many studies have reported high-throughput screening of small molecule libraries for drug development and toxicity assessment for liver diseases using iPSC-hepatocytes (59, 95, 96). Generation of organoids in several liver cell co-cultures yields a more sensitive cellular model than that constructed using single cell type. Recently, Broutier et al. (97) developed organoids representing the tumor structure and reflecting the expression profile of hepatocellular carcinoma, cholangiocarcinoma, and hepatocellular cholangiocarcinoma. These organoids open up new opportunities for drug testing and personalized medicine, and can be used for the generation of tumor bio-banks to be used as screening platforms (98). Additionally, “liver-on-a-chip” as a platform for drug development and toxicology testing can be used in pharmacokinetic and pharmacodynamic studies. In one study, liver organoids were generated using hepatocytes and cholangiocytes on a perfusable microcapillary chip (99). These organoids were then used to test the dose- and time-dependent hepatotoxic effects of acetaminophen. Thus, “organ-on-a-chip” represents a novel and valid platform for drug testing.

## Safety Evaluation of hiPSC-HLCs in Various Applications

While iPSCs have considerable applications in regenerative medicine, the genomic stability of these cells, such as the occurrence of genomic and epigenetic aberrations, copy number variation (CNV), and single-nucleotide polymorphisms (SNPs), is a matter of concern. Genomic aberrations in human PSCs (hPSCs) include abnormal karyotypes, such as recurrent trisomy of chromosomes 12, 17, or X, and aneuploidies of sub-chromosomal regions, such as duplications of the 12p, 17q, or 20q11.21 loci (100). Some studies have demonstrated that iPSCs, unlike somatic cells, contain regions of uniparental disomy (UPD), sharing the equivalent chromosomal and sub-chromosomal characteristics (101). These abnormalities may confer a selective advantage to certain genes during prolonged culturing.

In addition to genomic aberrations, other studies have focused on epigenetic aberrations, but the possibility of hiPSC application for complex diseases remains unknown (102). Epigenetic variations are often observed in different iPSC lines, and prolonged periods of culture (103) may affect disease modeling and clinical applications. For example, reactivating X-chromosome inactivation (XCI) and erosion of inactive X chromosome (Xi) silencing in female hiPSCs during iPSC reprogramming may trigger phenotypic and epigenetic changes in iPSCs, reducing their differentiation potential and increasing their tumorigenicity (104). Furthermore, local epigenetic variations in iPSCs, such as cellular memory and aberrant methylation loci, have been noted. Cellular memory leads to incomplete reprogramming, as DNA hypomethylation and histone modification at specific loci render iPSCs remain similar to the source cell. Further, iPSCs with cellular memory are susceptible to preferential differentiation into the cell type that they had been derived from (105–108). The iPSC-associated methylated loci contain certain imprinted loci and other genomic regions. Some alteration of genomic imprinted loci occurs during cell reprogramming or long-term culture (109–112). For instance, aberrant silencing of the *D1K1-Dio3* imprinted locus is functionally associated with the failure to generate iPSCs in mouse during reprogramming (113, 114). Methylome profiling has been used to detect differentially methylated regions (DMRs) in hESCs and hiPSCs (115). In the study, all hypermethylated CG DMRs in hiPSCs were recognized as reprogramming-induced aberrancies.

CNVs and SNVs can be introduced into iPSCs as they may already exist in source cell lines or be acquired during the reprogramming process. hiPSCs contain more CNVs than hESCs, source cells, and somatic cells (116). Therefore, it is possible that iPSCs are more susceptible to CNV generation during cell reprogramming. However, sequencing-based findings suggest the occurrence of few or no detectable *de novo* CNVs in iPSCs (117–121). By contrast, low-grade genetic mosaicism of CNVs in source cells was tracked to iPSC derivation. Hence, it is possible that low-grade genetic mosaicism of somatic cells is the major source of CNVs in iPSCs. In addition, several studies have discussed the potential relationship between SNVs and iPSC generation. Most studies revealed no specific functional enrichment of genes with SNVs in iPSCs (117, 120, 122, 123). Most iPSC-manifested SNVs appear to be randomly distributed in the genome and are functionally irrelevant to iPSC generation. Therefore, the safety of iPSCs for use in regenerative medicine still faces many challenges.

**Figure 3:**
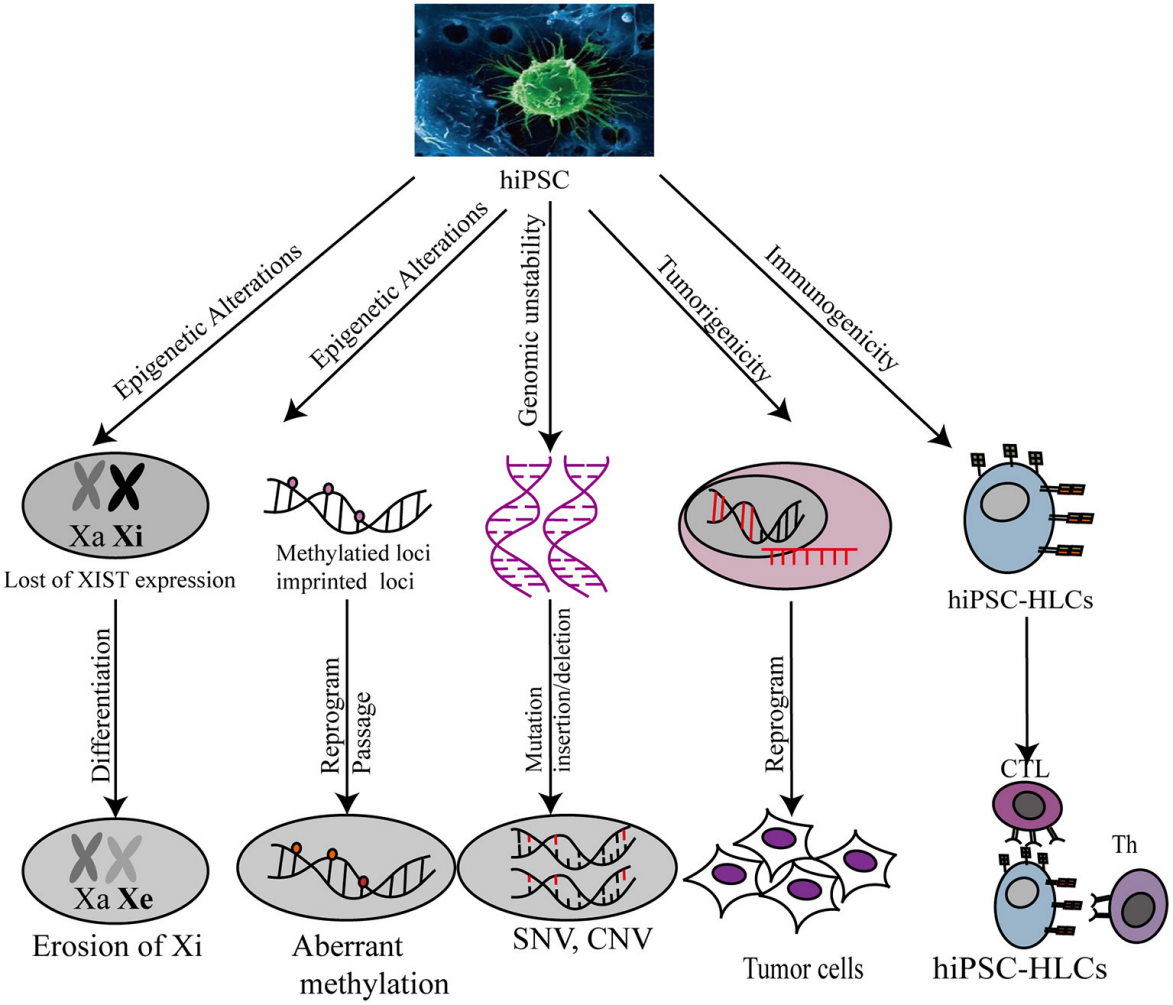
Safety evaluation of hiPSC-HLC in various applications. Xa, X chromosome activity; Xi, X chromosome inactivity; XIST, X-inactive specific transcripts; CNV, copy number variation; SNP, single nucleotide polymorphism.

Another safety concern has been raised regarding immunogenicity and tumorigenicity of the iPSC technology. Both genetic and epigenetic instability arising from iPSC reprogramming increases the risk of immunogenicity and tumorigenicity *in vivo* during iPSC-derived hepatic cell-associated therapy. Theoretically, the recipients of autologous iPSC-HLC transplants should not reject the transplants (124). However, in a teratoma mouse model, immune rejection in recipients was observed after iPSC transplantation (125). Undifferentiated PSCs, which possess the privilege property of immune tolerance because of low MHC-I antigen expression and the absence of MCH-II antigen expression, are expected to be less immunogenic than iPSCs (126–129). However, abnormal epigenetic differences between iPSCs and PSCs could contribute to the expression of immunogenic antigens during iPSC differentiation (128). Further, iPSC tumorigenicity poses a challenge for the development of individualized iPSC-HLC therapy. Pluripotency acquired by somatic cells upon reprogramming methods can increase genomic instability on the chromosomal and sub-chromosomal levels, contributing to the risk of tumorigenic transformation (130). Therefore, before the clinical development of iPSCs, current reprogramming technologies need to be optimized to minimize the occurrence of immunogenicity and tumorigenicity (Figure 3).

## Conclusion

**Figure 4:**
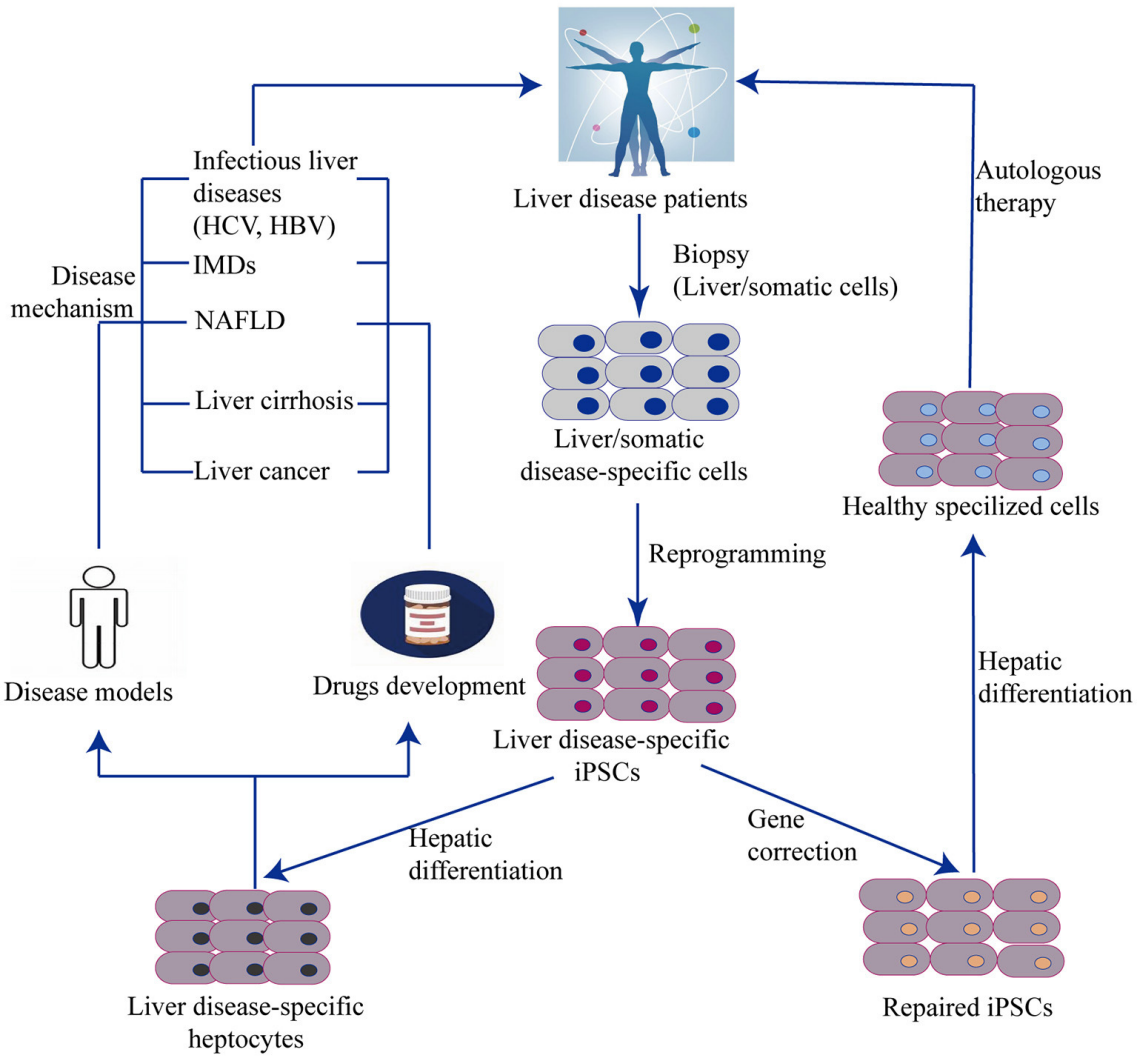
Generation of liver cells from iPSC and their applications. The scheme illustrates an overview of iPSC and organoid technology in relation to liver diseases. Disease specific iPSCs are generated by reprogramming technology from a biopsy of patients. One hand, iPSCs with disease–related gene mutation can be corrected by genome editing and then differentiated into functional disease-specific liver cells *in vitro*. These corrected cells are used to autologous cell therapy. Another hand, Non-corrected iPSC-derived disease-specific liver cells can be remodeled liver disease phenotypes, pathogenesis, and drug testing. IMD, Inherit metabolism disease; NAFLD, Non-alcoholic fatty liver disease.

Together with the burgeoning application of stem cell-based techniques, iPSC technology has been incorporated into new approaches such as -omics–related research, nuclear reprogramming, gene-editing technology, RNAi, tissue engineering, medical devices, high-throughput screens (HTS), and humanized chimeric animal models. iPSCs provide promising opportunities to study novel therapies for liver diseases using cell properties of self-renewal and differentiation for the generation of HLCs. Recent studies have demonstrated that iPSC-derived hepatocytes are applicable for *in vitro* studies of complex liver disorders, such as viral hepatitis, inherited metabolic disorders, non-alcoholic liver diseases, cirrhosis, and HCC (Figure 4). HLCs can also be used as cell therapy to repair and regenerate liver mass to treat liver disease and prolong patient survival. The genetic and molecular mechanisms underlying liver disorders are an emerging area of research. By studying iPSC-HLCs as a possible treatment option, researchers are able to gain valuable insight into *in vitro* disease modeling and personalized medicine.

Nevertheless, several limitations still exist that prevent the clinical application of iPSC technology. The protocols for reprogramming and differentiation of hiPSCs into HLCs should be optimized and standardized to increase the efficiency of inducibility and promote HLC maturation. The risk of potential tumorigenicity associated with genomic and epigenetic variations should also be assessed. In addition, to commercialize iPSCs for clinical applications, further investigation into the limitations and challenges of using iPSCs is needed. Very few scientific reports have tested HLC injection/transplantation in life-threatening models of congenital diseases. To the best of our knowledge, to date, no single study has been able to demonstrate correction of amino acid or neurotransmitter abnormalities by injecting iPSC-HLCs. Nonetheless, with rapid technological advances in stem cell therapy, hiPSCs are likely to become effective and safe treatment of liver diseases in the future.

## Author Contributions

LQ provided the concept of this manuscript and prepared the figures. LZ, KP, and XL drafted this manuscript and prepared the figures. SDWB, RN, SB, and YL revised the manuscript. All authors contributed to the article and approved the submitted version.

## Conflict of Interest

The authors declare that the research was conducted in the absence of any commercial or financial relationships that could be construed as a potential conflict of interest.

## Abbreviations

iPSCs: induced pluripotent stem cell
HLC: hepatic like cell
PHH: primary human hepatocytes; confidence interval
ESLD: end-stage liver disease
ESC: embryonic stem cells
IMD: metabolic disorder of liver
HCC: Hepatocellular carcinoma
NAFLD: non-alcoholic fatty liver disease
NASH: Non-alcoholic steatohepatitis
HBV: hepatitis B virus
HCV: hepatitis C virus
AATD: α1-antitrypsin deficiency
FH: familial hypercholesterolemia
EVs: extracellular vesicles
HSC: hepatic stellate cell
CSC: cancer stem cells
XCI: X-chromosome inactivation
CNV: copy number variations
SNV: single-nucleotide variations.
